# 

**Published:** 1844-04

**Authors:** 


### CONTENTS

OF NO. IV.

### ORIGINAL COMMUNICATIONS.

### ESSAYS AND CASES.

Art I.—Speculations on the facts presented in the Analytical Report on Mesmeric Somniloquism. By Daniel Drake, M.D. - - - - - - - 285

Art. II.—A Case in which the application of a small quantity of Strychina was followed by unpleasant effects. By W. L. Sutton, M.D., of Georgetown, Ky. 313

### REVIEWS.

Art. III.—Inquiries into the Frequency of Hernia, according to sex and age, and in relation to Population. By J. F. Malgaigne. - - - - - - 315

Art. IV.—The Principles and Practice of Medicine. By J. Elliotson, M. D., Cantab., F. R. S., &c. Edited by Nathaniel Rogers, M.D., &c., and Alexander Cooper Lee. With notes and additions: by Thomas Stewardson, M.D., Physician to the Pennsylvania Hospital. - - - - - - 340

SELECTIONS FROM AMERICAN AND FOREIGN JOURNALS.

Proximate causes of Hemorrhage. - - - - - 352
Mercury in Scrofulous Affections of the Lungs. - - 353
Veterinary Surgery. - - - - - - - - 353
On the Increasing Frequency of Cancerous Diseases. - 356
Veratria in Facial Neuralgia. - - - - - - 358
Advantage of Medicines in a Liquid Form. - - - 358
The Automaton with articulated voice. - - - - 359
Capsules of Cod-Liver Oil. - - - - - - 359
Compliment to the Memory of Sir Charles Bell. - 359
American origin of Syphilis. - - - - - - 360
Effects of a large dose of Quinine, adminstered by mistake. 362
Remarks on Italian Medicine. - - - - - - 363

ORIGINAL INTELLIGENCE.

Mesmerism. - - - - - - - - - - 368
Lunatic Hospitals. - - - - - - - - 369
The Burning-Wells of Kenawha. - - - - - 373
Anatomical Atlas. - - - - - - - - 375
Books Received. - - - - - - - - 376

